# Electrical protein detection in cell lysates using high-density peptide-aptamer microarrays

**DOI:** 10.1186/jbiol62

**Published:** 2008-01-31

**Authors:** David Evans, Steven Johnson, Sophie Laurenson, A Giles Davies, Paul Ko Ferrigno, Christoph Wälti

**Affiliations:** 1 School of Electronic and Electrical Engineering, University of Leeds, Leeds LS2 9JT, UK; 2 MRC Cancer Cell Unit, Hutchison/MRC Research Centre, Hills Road, Cambridge CB2 2XZ, UK; 3 Leeds Institute of Molecular Medicine, St James' University Hospital, Leeds LS9 7TF, UK

## Abstract

**Background:**

The dissection of biological pathways and of the molecular basis of disease requires devices to analyze simultaneously a staggering number of protein isoforms in a given cell under given conditions. Such devices face significant challenges, including the identification of probe molecules specific for each protein isoform, protein immobilization techniques with micrometer or submicrometer resolution, and the development of a sensing mechanism capable of very high-density, highly multiplexed detection.

**Results:**

We present a novel strategy that offers practical solutions to these challenges, featuring peptide aptamers as artificial protein detectors arrayed on gold electrodes with feature sizes one order of magnitude smaller than existing formats. We describe a method to immobilize specific peptide aptamers on individual electrodes at the micrometer scale, together with a robust and label-free electronic sensing system. As a proving proof of principle experiment, we demonstrate the specific recognition of cyclin-dependent protein kinases in whole-cell lysates using arrays of ten electrodes functionalized with individual peptide aptamers, with no measurable cross-talk between electrodes. The sensitivity is within the clinically relevant range and can detect proteins against the high, whole-cell lysate background.

**Conclusion:**

The use of peptide aptamers selected *in vivo *to recognize specific protein isoforms, the ability to functionalize each microelectrode individually, the electronic nature and scalability of the label-free detection and the scalability of the array fabrication combine to yield the potential for highly multiplexed devices with increasingly small detection areas and higher sensitivities that may ultimately allow the simultaneous monitoring of tens or hundreds of thousands of protein isoforms.

## Background

A comprehensive understanding of protein pathways in cellular processes and in disease states, as well as the identification of novel disease biomarkers, will require the ability to monitor tens or even hundreds of thousands of protein species in parallel. This represents a significant technological challenge that will involve the fabrication of functional high-density micrometer- or submicrometer-scale arrays, and much attention has been paid to the development of suitable techniques to enable such investigations [1-3]. While the problem of highly multiplexed monitoring is tractable at the level of DNA [4,5], the problem becomes significantly more complex when seeking to interrogate proteins, for which several alternatively spliced variants may be expressed from a single gene, with each variant then being subject to posttranslational modifications that regulate its activity and conformation. Thus, the development of the protein equivalent of the DNA microarray – the protein array – faces several challenges. The first of these is the identification of specific, high-affinity robust probe molecules that can bind each of the range of conformations that native proteins can adopt. A second is preserving these conformations for analysis, which will require the development of label-free sensing strategies. A third is the fact that strategies must be suitable for the detection of low-abundance proteins in complex biological solutions. Finally, the fabrication of such arrays will require a technology capable of immobilizing specific probe molecules with micrometer or submicrometer accuracy, as the total surface area required to present very large numbers of probes will dictate the minimum sample volume that can be investigated.

Currently, most protein microarrays are produced by dot printing and consist of surface-immobilized antibodies; interactions with sample ligands are detected optically [6]. However, the antibodies used are generally made against denatured, prokaryotically expressed proteins either by immunization of animals or selection *in vitro *from phage display libraries. Consequently, the antibodies predominantly recognize linear epitopes, which severely hinders their usefulness in applications to detect native proteins in complex biological mixtures. Antibodies are also relatively fragile molecules. Furthermore, when immobilized on a surface they often exhibit affinities for a number of different ligands, and hence lack specificity [7]. Alternative probe molecules, for example DNA and RNA aptamers [8-11], have been used in protein arrays, and although likely to be more robust than antibodies, they are also traditionally selected for binding to prokaryotically expressed proteins that may not be correctly folded, and will not be correctly posttranslationally modified. Another problem with current methodologies is that, although several label-free detection strategies have been discussed, including surface-plasmon resonance [12], mass spectroscopy [13], and atomic force microscopy-based techniques [14,15], the predominant method for the detection of probe-target interactions is based on fluorescent labeling of the target proteins. The fluorescent dyes are typically hydrophobic and may lead to conformational changes when covalently bound to a protein that will mask biologically relevant conformations [6]. Finally, the dot-printing techniques applied to conventional protein arrays offer resolutions of around 0.1 mm. Although the resolution of these printing and sensing techniques has improved in recent years, the feature sizes required for high-density protein arrays capable of handling extremely small sample volumes are beyond the scope of such systems. Alternative techniques, which enable the immobilization of probe molecules on surfaces such as metal electrodes with resolutions in the micro- or nanometer range, are required.

Here we offer solutions to each of these problems. First, we use robust, *in vivo *selected peptide aptamers as probe molecules to replace antibodies. Second, we use a novel molecular resist technology to functionalize different closely spaced gold electrodes with different peptide aptamers, resulting in feature sizes more than one order of magnitude smaller than current protein-array technologies. This functionalization technique is inherently scalable to submicrometer and nanometer feature sizes [16]. Third, we use an electronic, label-free method to detect binding events occurring between target proteins in solution and peptide aptamers immobilized on high-density electrode arrays. The array devices are fabricated using conventional silicon microfabrication technology, where miniaturization is well established. This, coupled with the scalability of the electrode functionalization technique, yields the potential for submicrometer, high-density functional devices with integrated readout technology capable of performing the many simultaneous measurements required for proteome-wide studies.

## Results and discussion

### Peptide aptamer design

Peptide aptamers are short peptide sequences presented and conformationally constrained in a robust, inert protein scaffold. They are selected *in vivo *from large libraries (as many as 10^9 ^unique peptide sequences), yielding binders with high and very specific affinities for selected targets [17,18]. The three-dimensional conformational constraint of the inserted peptide applied by the protein scaffold greatly increases the affinity of the aptamer for the target over that of an unconstrained peptide sequence [17-20]. They are distinguished from similar protein or peptide-based strategies by being selected in eukaryotic cells for binding to targets from large libraries using a yeast two-hybrid approach [21,22]. This maximizes the likelihood that the target protein is folded in the same way, and undergoes the same posttranslational modifications, as in its native environment, providing reliability and confidence in the specificity of detection.

**Figure 1:**
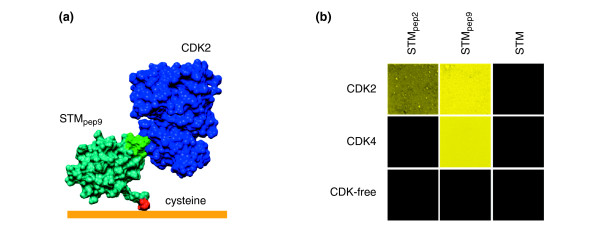
Peptide aptamer – CDK interactions. **(a)** Schematic diagram of STM_pep9_-CDK2 complex immobilized on a gold surface via conjugation between the SH group in a cysteine residue and the gold. **(b)** FRET analysis of STM_pep2_, STM_pep9 _and STM in solution on exposure to CDK2, CDK4 and CDK-free lysate. The peptide aptamers and lysate proteins were tagged with Alexa Fluor 488 and Alexa Fluor 555 fluorescent probes, respectively. The sample was excited at 488 nm and the resulting fluorescence from the Alexa Fluor 555 dye at 582 nm was measured. Yellow, strong interaction; dark yellow, medium interaction; black, no detectable interaction.

Traditionally, the *Escherichia coli *protein thioredoxin A (TrxA) has been used as a scaffold to present peptide aptamers [17]. However, many TrxA-based peptide aptamers are not expressed stably, which limits their use. In addition, the expression of TrxA in mammalian cells can lead to detectable phenotypes [23,24], indicating that the bacterial protein scaffold is able to interact with mammalian proteins, which would inevitably increase the background signal in any microarray format. We recently developed a new biologically inert protein scaffold (Stefin A triple mutant (STM)) that can present a wide range of different peptide sequences and has a simplicity and robustness that allows the production technique to be generic for all targets [25]. Here, we use peptide aptamers in STM to demonstrate label-free electronic transduction of biorecognition events occurring between human cyclin-dependent protein kinases (CDKs) as target proteins in solution and specific peptide aptamers immobilized on gold electrode arrays. Immobilization of the STM scaffold is achieved by the introduction of a single cysteine residue at the amino terminus, enabling attachment to gold electrodes via S-Au bonds (see [26] for further details). This cysteine residue is the only cysteine present in the scaffold and, in three dimensions, is located at the opposite side to the peptide insert (Figure 1a), thus separating the peptide insert from the surface.

In this study, we used two different peptide aptamers displayed by cysteine-modified STM, STM_pep2 _and STM_pep9_, where the subscripts refer to two different peptide inserts that both recognize cyclin-dependent kinase 2 (CDK2), but where only STM_pep9 _binds to cyclin-dependent kinase 4 (CDK4). Both CDK2 and CDK4 belong to a group of proteins involved in the regulation of the cell cycle; they are functionally related, yet have less than 46% sequence identity. STM_pep2 _and STM_pep9 _were generated by insertion of oligonucleotides encoding the CDK-interacting peptide sequences derived from the thioredoxin-based peptide aptamers of Colas *et al*. [17] into restriction sites in the open reading frame encoding the STM protein scaffold [25,27]. The binding of CDK2 and CDK4 to the peptide aptamers was confirmed *in vivo *using yeast two-hybrid interaction analysis ([25] and PKF, SJ, DE, SL, AGD and CW, unpublished work) and *in vitro *by fluorescence resonant energy transfer spectroscopy (FRET, Figure 1b) and dual polarization interferometry (DPI) [27]. The latter experiments showed that the affinity between surface-immobilized STM_pep9 _and CDK2 in a complex biological mixture compares well to typical values of *K*_D _for surface-immobilized antibodies [27]. These *in vitro *techniques also confirmed that the performance of the peptide aptamers is not affected by tethering the STM scaffold to a surface.

### Electrochemical detection of proteins

Electronic, label-free, on-chip detection of the peptide aptamer-target interactions is based on monitoring local changes in the impedance of the electrochemical double layer, which forms above the surface of a metal electrode when it is submerged in an electrolyte [28]. Any perturbation of this double layer, for instance by attachment of proteins to the electrode, alters the electrical properties of the layer. For example, the complex electrical impedance *Z*(*ω*) is a measure of the extent to which the charge transfer to and from the electrode is impeded by the surface-immobilized proteins. Hence, *Z*(*ω*) depends on the density, thickness and internal structure of the protein layer, and any alteration in this layer, such as the binding of a molecular target, potentially results in a measurable change of *Z*(*ω*) [29]. *Z*(*ω*) can be determined from the response of the system, i.e. the electrochemical current *I*, upon applying an ac electrochemical potential *Φ *of frequency *ω *to the electrode. Changes in *Z*(*ω*) manifest themselves in changes of the absolute impedance |*Z*(*ω*)| and its phase *φ*(*ω*), that is, the phase difference between *Φ *and *I*. We note that while |*Z*(*ω*)| scales with the electrode surface area, *φ*(*ω*) is independent of the electrode area, and changes in *φ*(*ω*), Δ*φ*(*ω*), therefore provide a reliable and reproducible measure of changes at the electrode surface, which will not be affected by variability in electrode surface area.

We used electrochemical impedance spectroscopy (EIS) to determine |*Z*(*ω*)| and *φ*(*ω*) as a function of frequency, *ω*. The measurements were performed in the presence of a redox probe (K_3_Fe(CN)_6_^4-/3-^) [29]. Gold electrodes functionalized with STM_pep9 _or STM were exposed to 45 μl of a solution containing about 200 ng/μl of purified, recombinant CDK2 (rCDK2) expressed in *E. coli *and subsequently rinsed with deionized water to remove any excess rCDK2.

**Figure 2:**
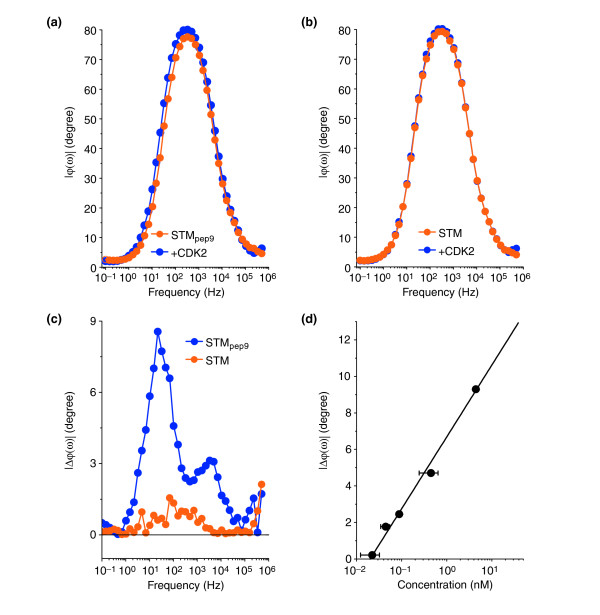
Electrochemical impedance spectroscopy of the interaction of STM_pep9 _with recombinant CDK2. **(a) **EIS *φ*(*ω*) data for a gold surface functionalized with STM_pep9 _(orange circles) and subsequently exposed to recombinant CDK2 (blue circles). **(b) **As (a) but following exposure of STM to rCDK2. **(c) **Phase shift (Δ*φ*(*ω*)) of STM_pep9 _and an STM layer on two independent gold electrodes following exposure to rCDK2. **(d) **Concentration dependence of the phase shift at 70 Hz. The solid line is a linear fit to the data (see text) provided as a guide to the eye.

Figure 2a,b show *φ*(*ω*) for STM_pep9_- and STM-functionalized electrodes, respectively, both before and after exposure to purified rCDK2. A shift in *φ *is observed upon rCDK2 binding to STM_pep9_, whereas no change was detected in the case of STM. This shift is more obvious when plotting the difference in the phase, Δ*φ*, before and after exposure to rCDK2 (Figure 2c). Whereas a pronounced peak in Δ*φ *is measured for STM_pep9_, no change in Δ*φ *is observed for STM. These results demonstrate that Δ*φ *provides a method to detect binding of the targets to the probe molecules immobilized on a gold electrode.

To determine the concentration dependence of the phase shift, Δ*φ*(*c*), gold electrodes were functionalized with STM_pep9 _and were exposed to 50 μl of phosphate buffer containing a range of concentrations of purified baculoviral CDK2 between 25 pM and 100 nM. The electrodes were subsequently rinsed in phosphate buffer to remove any excess CDK2 before *φ*(*ω*) was measured. The results are shown in Figure 2d from which a sensitivity limit of around 50 pM (approximately equal to 1.5 ng/ml CDK2) can be determined, which is in the clinically relevant range [7]. The phase shift is linear on a logarithmic concentration scale over more than three orders of magnitude. The solid line in Figure 2d represents a linear fit to the data.

### Detection of proteins in complex biological mixtures

In biologically and medically relevant samples, the proteins of interest are typically only present at low concentration and in complex mixtures of similar biological molecules. To assess the suitability of our sensing technique for the detection of proteins in such samples, we prepared gold electrodes functionalized with STM_pep9 _and STM, and exposed them to 35 μl of a whole-cell lysate of CDK2-expressing yeast cells. Following exposure to yeast lysate, the devices were thoroughly washed to remove any non-specifically bound material.

**Figure 3:**
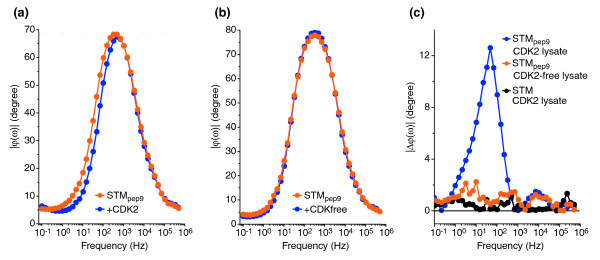
Electrochemical impedance spectroscopy of the interaction of STM_pep9 _with CDK2-expressing cell lysate. **(a) **EIS *φ*(*ω*) data for a gold surface functionalized with STM_pep9 _(orange circles) and following exposure to CDK2-expressing yeast lysate (blue circles). **(b) **As (a) but following exposure of STM_pep9 _to a CDK-free yeast lysate. **(c) **Data from (a) and (b) plotted as the phase shift (Δ*φ*(*ω*)) for independent electrodes functionalized with STM_pep9 _and exposed to either a CDK2-expressing yeast lysate (blue line) or a CDK2-free lysate (orange line). The black line represents the Δ*φ*(*ω*) of an STM-functionalized gold electrode following exposure to CDK2-expressing yeast lysate. A phase shift is only detectable upon exposure of STM_pep9 _to CDK2, indicating that yeast proteins neither interact nonspecifically with the peptide aptamers nor specifically or nonspecifically with the electrode surface.

The phase *φ*(ω) of the complex impedance measured for the different devices is shown in Figure 3. Whereas a distinct shift in *φ*(ω) is observed between 1 and 10^3 ^ Hz for STM_pep9 _exposed to CDK2 lysate (Figure 3a,c), there is no change in *φ*(ω) across the whole frequency range investigated for STM exposed to the different aliquots of the same lysate (Figure 3c). Given that STM_pep9 _and STM differ only in the presence (or absence) of the peptide aptamer insert, the dramatic difference in impedance characteristics following exposure to the lysate must be related to an interaction with STM_pep9 _through the peptide insert. To confirm that this response is related to the formation of the CDK2-STM_pep9 _complex, rather than to binding with other species in the lysate, we exposed a series of STM_pep9 _functionalized electrodes to lysate of yeast cells that were not expressing CDK2 (Figure 3b). The lack of a shift in phase following exposure to this CDK-free yeast lysate (see Figure 3c) confirms the specific affinity of STM_pep9 _for CDK2. The protein complexity of a yeast lysate (comprising proteins expressed from approximately 4,000 open reading frames at any given time) compares favorably with that of medically relevant biofluids, such as urine (1,400 individual proteins [30]), and serum or plasma (with estimates of 1,175 [31] to 3,700 [32] individual proteins). These results demonstrate the ability of our peptide aptamer sensor to unambiguously detect target-aptamer binding from such samples.

### Multiplexed detection of proteins using high-density microarrays

Many future applications in biology (such as systems analysis of protein interactions) and medicine (such as personalized medicine protocols) will rely on the ability to investigate large numbers of proteins simultaneously. Very often the available sample volume is likely to be limited, particularly in the case of biopsies from patients. Therefore, high-density arrayed systems – that is, arrays with very small and very closely packed sensors – are required. Conventional technologies for generating protein arrays are generally based on dot-printing strategies with resolutions of the order of 0.1 mm or more. High-density arrays, with submicrometer or even micrometer feature sizes, are beyond the scope of these approaches. To enable the immobilization of different peptide aptamers on different electrodes spaced only a few micrometers apart, we developed a process inspired by our previous work [16]. This enables the selective functionalization of individual microelectrodes of an array with resolutions at least an order of magnitude better than that achieved by conventional techniques.

**Figure 4:**
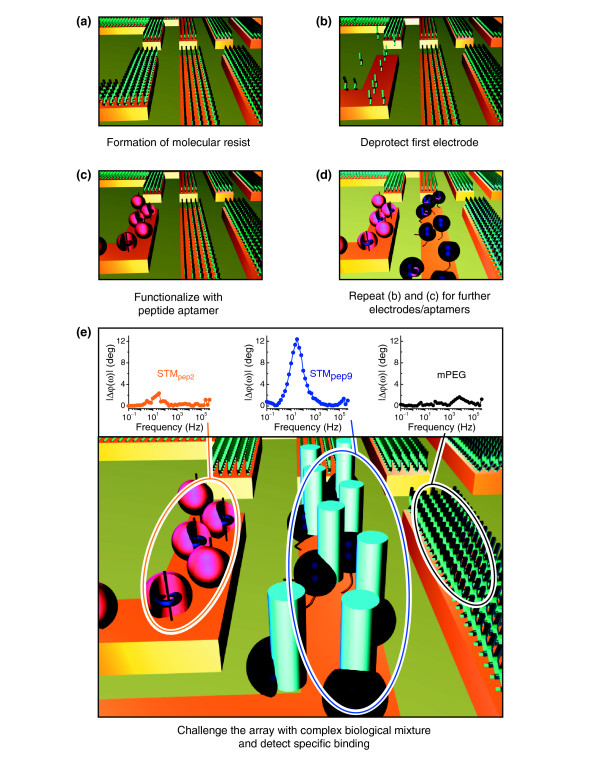
Schematic diagram showing the use of a molecular mask for selective functionalization of a microelectrode array. **(a) **All microelectrodes are initially protected from functionalization by a protein-inhibiting mPEG monolayer. **(b) **This molecular mask can be released by electrochemical means. The left microelectrode is addressed, while all neighboring microelectrodes are actively prevented from electrochemical desorption via a second potentiostat. **(c) **The bare gold microelectrode surface is functionalized with the required peptide aptamer. **(d) **By repeating this cycle it is possible to independently functionalize multiple microelectrodes with different proteins within a single device. **(e) **The formation of a protein-protein complex following exposure to a complex biological solution results in a measurable change (phase shift) in *φ*(*ω*) (central microelectrode). *φ*(*ω*) remains constant for all microelectrodes in which target-specific binding does not occur (left and right microelectrodes), thus enabling unambiguous identification of protein targets within the biological sample.

Selective functionalization of the microelectrodes with different peptide aptamers was achieved through the molecular masking process illustrated in Figure 4. The microelectrode arrays comprised ten individually addressable gold microelectrodes of 20 μm width and separated by 15 μm, which were first coated with a methyl-terminated poly(ethylene-glycol)_6_-thiol (mPEG, Polypure, Oslo, Norway) layer that prevents nonspecific binding of proteins during microelectrode functionalization. The thiol-modification of the mPEG not only allows the spontaneous formation of a molecular monolayer on the gold microelectrodes through the Au-S bond, but also provides a means for removing the masking layer from any individual microelectrode through reductive cleavage of this bond [33,34]. The quality of the resulting mPEG layers was verified using water contact-angle measurements and X-ray photoelectron spectroscopy, and the effectiveness of protein inhibition was confirmed by fluorescence spectroscopy (data not shown). After formation of the mPEG layer, the arrays were soaked for 1 hour in deionized water to remove residual ethanol and to form a water layer around the mPEG, which is believed to be crucial in the inhibition of protein binding [35].

**Figure 5:**
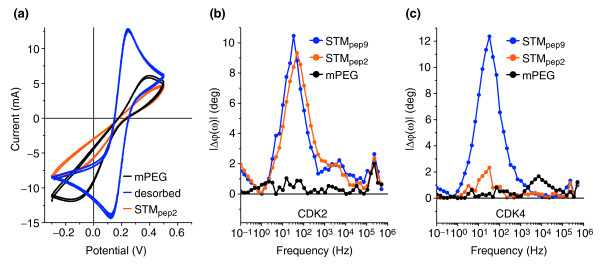
Multiplexed detection of proteins in cell lysate using high-density microarrays. **(a) **Cyclic voltammogram of an individual microelectrode protected with an mPEG protein-inhibiting layer (black line), and following electrochemical desorption of the mPEG monolayer (blue line), and after functionalization with peptide aptamer STM_pep2 _(orange line). **(b) **Δ*φ*(*ω*) of the complex impedance for microelectrodes functionalized with mPEG (black circles), STM_pep2 _(orange circles) and STM_pep9 _(blue circles) following exposure to a lysate containing CDK2. **(c) **As (b) but following exposure to cell lysate containing CDK4.

The mPEG molecular mask was selectively removed from an individual microelectrode by applying an electrochemical potential of -1.4 V versus Ag/AgCl for 120 seconds using a potentiostat and identical buffer conditions to those used in EIS measurements (Figure 4b). Owing to the small spacing between the microelectrodes, the electric fields generated during desorption may conceivably influence the electrochemical potential of neighboring microelectrodes, potentially disturbing the blocking layer. To prevent this while allowing scalability to smaller microelectrode geometries, a second potentiostat was used to hold the potential of the neighboring microelectrodes at -0.2 V versus Ag/AgCl during the desorption process. The efficacy of desorption is monitored by cyclic voltammetry (Figure 5a). Having desorbed the mPEG molecular mask, the bare gold microelectrode can be functionalized with the desired protein by incubating the device in 35 μl protein solution overnight in a sealed humid environment (Figure 4c). The adsorption of the protein, and the effectiveness of the mPEG monolayers for masking deposition on protected microelectrodes, is confirmed using cyclic voltammetry and EIS. This process can be repeated to functionalize further microelectrodes with different proteins (Figure 4d).

Both STM_pep9 _and STM_pep2 _show an affinity for CDK2, whereas only STM_pep9 _showed an affinity for CDK4 (Figure 1b). We exploit this difference in functionality to demonstrate our multielectrode array sensor's ability to discriminate between binding events occurring on differently functionalized microelectrodes, fabricated on a single device. Two separate, nominally identical array devices with adjacent individual microelectrodes functionalized with the two different peptide aptamers, STM_pep9 _and STM_pep2_, were challenged with either CDK2- or CDK4-expressing lysate. The EIS results are shown in Figure 5b,c. Shifts in *φ*(*ω*) are observed for both the STM_pep9_- and STM_pep2_-functionalized microelectrodes following exposure to the CDK2-expressing yeast lysate, as expected from the FRET data (Figure 1b). Conversely, on exposure to CDK4-expressing yeast lysate, a shift in *φ*(*ω*) of similar magnitude was only observed for the STM_pep9 _functionalized microelectrode. The lack of response following exposure of STM_pep2_-functionalized microelectrodes to CDK4 indicates the high selectivity of the functionalization process. In all cases, *φ*(*ω*) remains constant for those microelectrodes coated with mPEG, confirming the efficacy of the inhibiting layer.

## Conclusion

In conclusion, we have demonstrated an electronic, label-free, array-format detection scheme for targeted proteins at clinically relevant concentrations from complex biological mixtures. It is based on *in-vivo*-selected peptide aptamers as probe molecules and a method for the individual functionalization of closely spaced electrodes with different peptide aptamers. Arrays with micrometer-scale dimensions have been implemented using these techniques. The electronic detection, fabrication and functionalization methods are inherently scalable, enabling the generation of very high-density microelectrode arrays. Such arrays may ultimately offer the possibility of protein chips providing massively parallel, label-free analysis of protein-protein interactions, and may eventually permit the analysis of multiple protein interactions simultaneously at the level of a single cell.

## Materials and methods

Unless otherwise stated, all chemicals were sourced from Sigma, Gillingham, UK.

### Peptide aptamers, yeast lysate, recombinant and viral CDKs

The peptide aptamers were generated and immobilized on gold electrodes as described in [26,27]. The yeast lysate was prepared as detailed in [18,27]. Purified activated baculoviral CDK2 was sourced from Cell Signaling Technology (Danvers, MA).

### FRET experiments

STM and STM_pep9 _were labeled with Alexafluor 488 TFP ester (Molecular Probes, Invitrogen, Carlsbad, CA). Ten microliters of protein (approximately 2 mg/ml) were diluted with 10 μl 1 M NaCO_3 _aqueous solution, after which 5 μl Alexafluor dissolved in dimethyl sulfoxide (DMSO) was added. This mixture was left to incubate for 1 h, after which 1 μl Tris HCl was added to bind unbound dye. The solution was then filtered through a protein-desalting column (Pierce, Rockville, IL) to remove excess fluorophores. The CDK lysates were also dyed in the same way but using Alexafluor 555 NHS ester (Molecular Probes).

### Microelectrode array fabrication

Each electrode array structure comprises two opposing rows of five individually addressable electrodes, each 20 μm wide and separated by 15 μm. The electrode structure was fabricated using double lift-off photolithography as follows. N-doped silicon (100) substrates 30 μm thick with a thermally grown 500 nm oxide (Compart Technology, Peterborough, UK) were cleaned in piranha solution (70% H_2_SO_4_:30% H_2_O_2_) at 100°C for 5 min. The wafer was baked at 150°C for 60 min before polymethyl methacrylate (PMMA, MW 950 kDa, 4% in anisole) was spun on at 1,500 rpm for 40 sec. Following a 90-min bake step at 150°C, Shipley 1805 photoresist was spun onto the PMMA at 5,000 rpm for 40 sec and baked on a hotplate at 115°C. The wafer was then cleaved, and individual sections were exposed under 8 mW/cm^2 ^ultraviolet light (UV) in a mask aligner for 4 sec and developed in MF319 photoresist developer for 60 sec. The chip was rinsed in deionized water (Millipore, 18.2 Ωcm) and transferred to a UV ozone cleaner where it was exposed to deep UV for 16 min to expose the PMMA and harden the upper photoresist layer. Following this treatment, the PMMA was developed in 3:1 isopropanol:methyl isobutyl ketone for 20 sec and rinsed in isopropanol before the exposed surface was cleaned in 1:5 HCl:H_2_O. Finally, 15 nm Cr and 50 nm Au were evaporated onto the wafers, and lifted off using acetone for 30 min. Individual devices were then scribed out, and the wafer cleaved. The devices were mounted in ceramic packages with Araldite, wire bonded and cleaned in piranha solution before functionalization.

### Electrode functionalization

Gold electrodes were functionalized with peptide aptamers by exposure to 35 μl of either STM_pep2_, STM_pep9 _or STM in a PBS buffer (pH 7.3) for 18 h at room temperature. The devices were subsequently exposed to the solution containing the target proteins and then rinsed with deionized water (18.2 Ωcm, Millipore) to remove any excess target solution. Coating the gold electrodes with mPEG was achieved by immersing the devices in a 10 mM ethanolic solution for 96 h.

### Electrochemical impedance spectroscopy (EIS)

EIS measurements on individual gold electrodes, where |*Z*(*ω*)| and *φ*(*ω*) are determined as a function of frequency between 0.1 Hz and 500 kHz, were carried out using a custom built three-electrode electrochemical cell with an electrolyte volume of 50 ml, and a Princeton Applied Research VSP bipotentiostat/impedance spectrometer in a common-electrode-to-ground configuration. The gold electrode was used as the working electrode, and a 50-mm-long high-purity Pt wire (Agar Scientific, Stanstead, UK) was fully immersed in the electrolyte and used as the common electrode. The reference electrode was a Ag/AgCl double-junction ceramic wick electrode (VWR, Lutterworth, UK). The electrolyte consisted of 100 mM phosphate buffer pH 7.0 containing 10 mM K_3_Fe(CN)_6_^4-/3- ^redox probe which decreases the impedance of the system by increasing the measurable quantities and hence improves the sensitivity of the system [29]. All electrochemical potentials are reported against a Ag/AgCl reference electrode. Errors in the accuracy of the phase-shift measurements can be determined from the cumulative error of the response to CDK-free phosphate buffer and the measurement error revealed through repeating the readings; the overall error in the phase shift is estimated to be ± 0.5°.

### Concentration dependence and sensitivity limit measurements

Concentration dependence and sensitivity limits were measured by exposing the gold electrodes, functionalized with STM_pep9_, to phosphate buffer solutions containing various concentrations of commercial purified activated CDK2 (Cell Signaling Technology). The STM_pep9_-functionalized electrodes were placed in 100 mM phosphate buffer containing 10 mM redox probe (K_3_Fe(CN)_6_^4-/3-^), and the impedance measurements were taken repeatedly to confirm stability. The redox buffer was then removed, and the chip rinsed in phosphate buffer without redox probe, and incubated in pure phosphate buffer for 60 min, after which impedance measurements were repeated to confirm the zero response of the surface. The gold electrode was then exposed for 1 h to 50 μl phosphate buffer containing the required concentration of CDK. Final impedance measurements were taken after the gold electrodes were rinsed in phosphate buffer, transferred to the measurement buffer (phosphate buffer containing the redox probe), and the system allowed to equilibrate. All buffers were filtered through a 0.22 μm filter before use. Each measurement was repeated three times to confirm the reading, and repeated sets of measurements were made at 30-min intervals to ensure stability. The accuracy in the concentration of the CDK solutions was determined by the accuracy of the pipettes used and the repeated dilution steps from an initial 100 ng/μl solution. Errors in the accuracy of the phase-shift measurements were calculated as above and the corresponding error bars are indicated on Figure 2d.

### Cyclic voltammetry (CV) experiments

The efficacy of the desorption of the molecular masking layer and the subsequent adsorption of the proteins was monitored using cyclic voltammetry (CV). These experiments were carried out in the same buffer conditions as the EIS measurements (100 mM phosphate buffer pH 7.7 with 10 mM K_3_Fe(CN)_6_^4-/3- ^redox probe) and at scan rates of 62 mV/s. The CV curve of the mPEG-coated gold electrode is shown in Figure 5a. Upon desorption of this layer, the peak separation on the voltammogram decreases from 425 mV to 100 mV, typical of a gold surface with this redox probe [29]. Following the adsorption of cysteine-modified STM_pep2 _onto the exposed gold electrode, the peaks in the CV curve decrease and the peak separation increases, indicating the successful binding of the peptide aptamer to the surface.
